# Evaluation of the Potential of Metakaolin, Electric Arc Furnace Slag, and Biomass Fly Ash for Geopolymer Cement Compositions †

**DOI:** 10.3390/ma16072741

**Published:** 2023-03-29

**Authors:** Tomás Archer de Carvalho, Florindo Gaspar, Ana C. Marques, Artur Mateus

**Affiliations:** 1 Centre for Rapid and Sustainable Product Development, Polytechnic of Leiria, 2430-028 Marinha Grande, Portugal; 2 Centre for Rapid and Sustainable Product Development, School of Technology and Management, Polytechnic of Leiria, 2430-028 Marinha Grande, Portugal; 3 CERENA, Chemical Engineering Department, Instituto Superior Técnico, Universidade de Lisboa, 1049-001 Lisboa, Portugal

**Keywords:** geopolymerization, geopolymer cement formulation, circular economy, inorganic industrial waste

## Abstract

The widespread use of geopolymer cement (GPC) has been hindered by a lack of scientific knowledge that still exists regarding its synthesis process. Key points, such as the release of aluminosilicate species from the raw materials and its link to the properties of GPC, have still not been completely studied. As a result, most of the GPC formulations covered in the literature are based on precursors’ elemental analysis using XRF (X-ray Fluorescence), or other equivalent analysis methods, and consider that the total aluminosilicate content of the precursors is available for participating in the geopolymerization process, which seems very unlikely. In this study, the amounts of aluminate and silicate species released from metakaolin (MK), electric arc furnace slag (EAFS), and biomass fly ash (BFA) in alkaline dissolution tests were determined by simple spectrophotometric methods. It was found that MK yields the highest aluminosilicate dissolution amount, about 2.1 mmol of silicate + aluminate per gram of MK, while EAFS and BFA yield about 0.53 and 0.32 mmol/g precursor, respectively. These results were used to estimate the total amounts of dissolved aluminosilicates in a series of GPC mortars prepared from these raw materials, which were thereafter subjected to mechanical tests. It was shown that the mortars’ compressive strength (which ranged from 1 to 63 MPa) is linearly correlated with their estimated total amount of dissolved aluminosilicates, with the best linear fit yielding a coefficient of determination above 0.99. It was concluded that by using the results of the dissolution tests, the estimation of compressive strength is greatly improved when compared to using the elemental analysis obtained by XRF, which yields a coefficient of determination of 0.88 and a larger dispersion of data points. The results reveal the usefulness of this simple method for evaluating the potential of inorganic industrial waste streams as precursors for GPC.

## 1. Introduction

The intense global consumption of raw materials is expected to continue and is estimated to double over the next four decades, resulting in an astonishing 70% increase in annual waste generation by 2050 [1]. Inorganic industrial waste, such as unused slag and fly ash, is commonly disposed of in landfills, which is an environmentally and economically unsustainable option, as existing landfills are reaching exhaustion and landfill taxes have increased in recent years [2]. The application of a circular economy paradigm, in which, e.g., virgin raw materials are replaced by byproducts or waste streams, is therefore a key factor in the industry’s path towards sustainability, enabling a critical reduction in extracted materials, generated waste, and greenhouse gas emissions.

In this context, the geopolymerization synthesis process has shown the potential to incorporate a significant proportion of inorganic byproducts or waste streams into the formulation of new and added-value materials, such as geopolymer cement (GPC). GPC can be described as a synthetic aluminosilicate cement and is produced by reacting a solid source of aluminosilicates in powder form with an aqueous alkaline solution, typically a hydroxide or silicate solution. GPC is comparable to ordinary Portland cement binders, namely in terms of the mechanical performance that can be obtained, and has the added environmental advantage of being able to recycle a wide variety of inorganic byproducts or waste streams as its main constituents (the source of aluminosilicates), resulting in a significantly lower carbon footprint [3,4,5,6,7]. The three most common raw material classes used in GPC synthesis are calcined clays (e.g., metakaolin) [6,8,9], fly ashes (e.g., coal fly ash) [9,10,11,12], and slags (e.g., metallurgical blast furnace slag) [9,11,13], all possessing pozzolanic activity. Apart from the most commonly used MK, FA, and GGBFS, many other byproducts and industrial wastes containing considerable amounts of reactive silica and alumina might be used as raw materials in the geopolymerization process. Among others, materials such as glass waste [14,15], ceramics waste [16,17,18], mine tailings [19,20], waste from natural stone processing [21,22], and rice husk ash [23,24] are currently being studied for possible integration in GPC formulations.

Despite the potential of GPC as an eco-friendly binder, the understanding of its synthesis process and technology is far from being complete, hindering its widespread use as an alternative to more conventional binders associated with a larger environmental footprint. In fact, the complex physico-chemical mechanisms on the basis of the geopolymerization process are still under study [5,8,12], but there has been agreement on the importance of the initial alkaline dissolution of raw material particles, which releases aluminate and silicate species into solution according to the following simplified processes [25]:

Al_2_O_3_ + 3H_2_O + 2OH^−^ → 2[Al(OH)_4_]^−^ (aluminate)

SiO_2_ + 2OH^−^ → [SiO_2_(OH)_2_]^2−^ (silicate)

Aluminate and silicate species, which are recognized as the most important chemical species in the GPC synthesis process [9,25], will therefore begin to accumulate in solution, reaching a point where they are able to recombine and reorganize, forming an aluminosilicate network that will progressively increase in extension and connectivity, allowing the material to set and harden [6,25]. In this respect, knowledge of the rate and extension of dissolution of aluminate and silicate species from the raw materials is critical, since the elemental composition alone will not enable a suitable characterization of the geopolymerization process, due to differences in phase reactivity and other physical and chemical effects [26,27]. In fact, the likelihood of the total silica and alumina content of the raw materials being involved in the geopolymerization reactions is very low [28,29]. Taking this into account, an estimation of the dissolved amounts of silicate and aluminate species is important in designing the GPC mix because these seem more likely related to the actual amounts taking part in the formation of the GPC matrix than the total SiO_2_ and Al_2_O_3_ present in the precursors [30]. However, most published works on GPC mix design have relied on the total oxide content reported in the precursors’ chemical analysis to calculate chemical ratios and mix proportions [30,31,32,33,34,35,36]. In fact, only a few recent research examples have explicitly investigated the relationship between the properties of the obtained GPC and the measured dissolution extent of silicates and aluminates from the precursors used [37,38].

In this study, the alkaline dissolution process of metakaolin (MK), biomass fly ash (BFA), and electric arc furnace slag (EAFS) is investigated. MK is a powdered material obtained by the calcination of kaolin clay. It is estimated that the production of kaolin in Portugal is currently around 320 kton/year [39] and that each ton of kaolin can eventually yield 840 kg of MK [40]. BFA is a solid waste stream produced during the combustion process in biomass power plants and is collected as dust from the gaseous stream exiting the furnace. In Portugal, the produced amount of BFA is expected to reach around 135 kton/year [41]. EAFS is a solid non-metallic waste material that forms during the recycling of scrap steel in an electric arc furnace. After some processing steps (crushing, metallic fraction separation, sieving) this material can be used, e.g., as an artificial aggregate [42,43,44]. Currently in Portugal, the production of treated EAFS is estimated at about 250 kton/year [45]. A 10 M sodium hydroxide (SH) solution is used as the alkaline medium and simple spectrophotometric methods are employed to measure the concentration of silicate and aluminate species dissolved from these precursors [37]. These results were then correlated with the chemical composition of the precursors and with the mechanical strength achieved by the resulting GPC mortar specimens prepared from these materials, with the aim of establishing a simple method for assessing the potential of such raw materials for the production of GPC mortars.

## 2. Materials and Methods

The general formulation of GPC mortars used in this study was:

- Precursor mix, comprised of one or more of the three aluminosilicate-containing raw materials MK, BFA, and EAFS.;
- Sand, used as fine aggregates;
- Alkalination or activation mix, comprised of a combination of sodium silicate (SS) and SH solutions, adjusted with additional water or SH pellets.

### 2.1. Precursors, Aggregates, and Alkalination Mix

**Figure 1:**
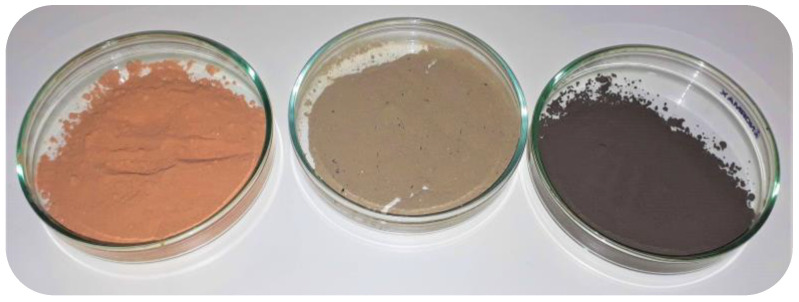
Used GPC precursors. From left to right: MK, BFA, and ground EAFS.

In the present study, MK, BFA, and EAFS were used as the main aluminosilicate-containing raw materials; i.e., the precursors of the geopolymerization process (Figure 1).

**Figure 2:**
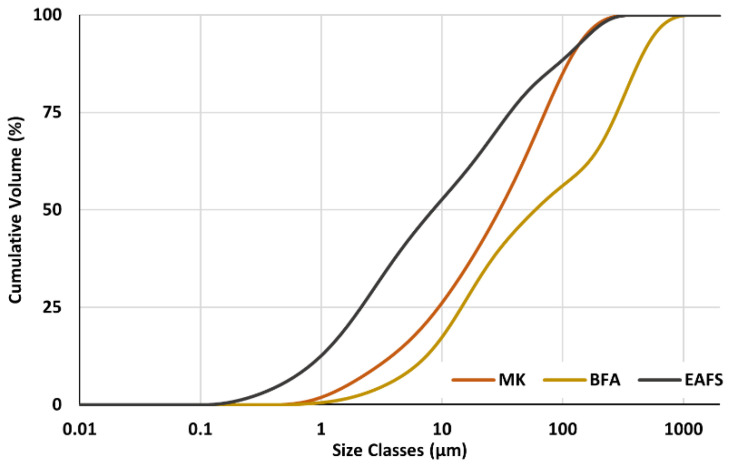
Cumulative PSD curves for GPC precursors.

MK used in this research was supplied by Mota Ceramic Solutions (Alvares, Portugal), while the used BFA originates from a plant of Greenvolt (Lisboa, Portugal), where it was separated from the gaseous stream by bag filters. Both MK and BFA are originally powdered materials and were used as received. As for EAFS, the material was supplied by Harsco Environmental (Maia, Portugal) and was composed of large granules (as large as 5 cm, in our case) that are not suited for use in the geopolymerization process. For this reason, the original material was ground in a laboratory mill (MGS, Rapid Mill MGS1800/2) to produce the fine powder needed for the GPC preparation. The specific gravity of MK, BFA, and EAFS, provided by the suppliers, is 2.4, 2.3, and 3.5, respectively. The particle size distribution (PSD) curves and the main PSD parameters for each used precursor, determined in a Masterziser 3000 laser diffraction particle size analyzer (Malvern Panalytical, Malvern, UK), are shown in Figure 2 and Table 1, respectively.

D10, D50, and D90 correspond to the particle size, below which 10%, 50%, and 90% of the sample volume is found, respectively.

The chemical composition of the precursors was determined by X-ray Fluorescence. For the case of MK, the composition was given by the supplier, whereas for BFA and EAFS, a Zetium XRF spectrometer (Malvern Panalytical), equipped with a 4 kW Rhodium X-ray tube, was used for the measurements. The main oxide proportions are reported in Table 2.

**Figure 3:**
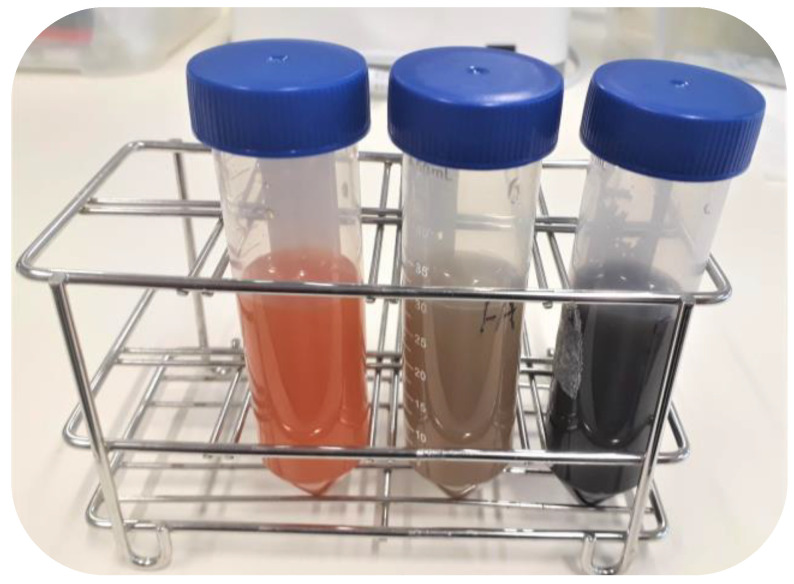
Suspension of GPC precursors resulting from 120 min of reaction time with 10 M sodium hydroxide solution. From left to right: MK, BFA, and EAFS.

The amount of dissolved aluminate and silicate species from the precursors in alkaline medium or, in other words, their potential reactivity towards the geopolymerization process was assessed by an alkaline dissolution test carried out as follows. A 10 M SH solution was prepared, dissolving SH pellets (Sharlau, ACS) in deionized water, and used as the alkaline medium or alkalination solution at a ratio of 200 mL/g of precursor. The resulting suspension was allowed to react for 120 min under controlled agitation, at room temperature (Figure 3).

Thereafter, the suspension was centrifuged and the liquid phase (comprised of silicates and aluminates in alkaline solution) was decanted and neutralized to a pH ≈ 7 with hydrochloric acid (Sigma-Aldrich, St. Louis, MI, USA, fuming, ≥37%). The concentration of silicate and aluminate species in the resulting solutions was then determined by visible spectrophotometric methods—the silicomolybdic acid method (SMA) [46] and the Eriochrome Cyanine R (ECR) method [47], respectively—using a SPECTROstar^Nano^ spectrophotometer from BMG LABTECH (Ortenberg, Germany). Sodium metasilicate (Sigma-Aldrich) and hydrated aluminum nitrate (Sigma-Aldrich, aluminum nitrate nonahydrate, ≥98%) were used for preparing the aqueous solutions for the calibration of the SMA and ERC methods, respectively.

Using this method, the total silicate and aluminate dissolved from the solid precursor into an alkalination solution—that, thus, becomes available for the geopolymerization process—could be estimated in terms of mol (SiO_2_ + Al_2_O_3_) per unit weight of raw material.

**Figure 4:**
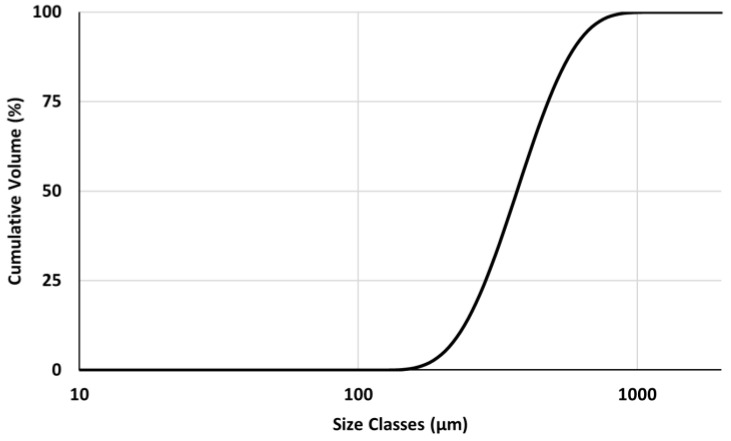
Cumulative PSD curves for sand used as fine aggregates.

In all GPC mortars, natural washed river sand with a specific gravity of 2.6, supplied by SACT, Lda. (Alpedriz, Portugal), was used as fine aggregate. The PSD cumulative curve and the main PSD parameters of sand, determined in a Mastersizer 3000, are presented in Figure 4 and Table 3, respectively.

The alkalination mix solution was comprised of varying proportions of SS solution (sodium silicate D40, supplied by Quimialmel, Lda. (Albergaria-a-Velha, Portugal)), SH solution (32% (38º BE), supplied by Ângelo Coimbra, S.A. (Maia, Portugal)), water, and solid SH (Sharlau, pellets, ACS).

### 2.2. Geopolymerization Parameters

GPC mortars were prepared with the abovementioned precursors, sand, and alkalination mix in six different formulations, and so that the performance of the different mortars could be compared, some of the most important geopolymerization parameters were fixed, as shown in Table 4. In order to obtain the desired ratios for all formulations, the alkalination mix had to be adjusted in each one, by varying the proportions of sodium silicate and sodium hydroxide solutions and correcting it with the addition of supplementary water or solid sodium hydroxide (see Table 5).

SiO_2_/Al_2_O_3_ is the ratio between silica and alumina in precursors and will affect the resulting GPC structure. To obtain a geopolymer material suited for cement-like applications, a reference value of SiO_2_/Al_2_O_3_ between 3.3 and 4.5 in the actual geopolymer structure should be respected [48]. Therefore, in this study, the used ratio of 4.4 is based on the results of reactivity tests instead of XRF analysis, since, as it will be shown later, the amount of silicates and aluminates released by the precursors (in other words, the reactive portion) is only a modest fraction of the total amount they contain as measured by XRF.

H_2_O/Na_2_O is the ratio between total water and total sodium oxide in solution and represents a measure of the pH used in the process. The pH of the alkalination solution will affect the dissolution process of aluminosilicates from precursors—generally, the higher the pH (lower H_2_O/Na_2_O), the higher the extent of dissolution of aluminosilicates [48] that will, therefore, be available for recombining and form the geopolymer matrix.

Water to solids (W/S) is the ratio between total water and total solids in the mortar (including precursors, aggregates, and dissolved solids in the alkalination solution). It will affect the consistency and workability of the GPC mortar and its ability to cure fast, as well as the final achieved mechanical strength [48]. Since the consistency and workability of a mortar depend on many factors, the used fixed value of W/S = 0.15 was chosen based on initial trials to adjust these parameters to a satisfactory level.

Aggregates to precursors (Agg/P) is the ratio between aggregates and precursors used in the mortar formulation. Since aggregates are assumed to be essentially inert, this ratio will determine the proportion of solid material available for taking part in the geopolymerization reaction. The higher the ratio, the lower the expected mechanical performance of the mortar, since there will be less binding material. In the present study, the fixed value of Agg/P = 2.0 was selected based on the typical range reported in the literature (between 1.0 and 3.0) for GPC mortars cured in room conditions [14,15,16,17,21,49,50,51,52,53].

### 2.3. Mortar Mixing, Casting, Curing, and Mechanical Testing

The mortar mixing procedure was carried out in a (Controls, 65-L0502) by first adding the weighted precursors and alkalination solution into the mixer and mixing for 120 s at 140 rpm, with a pause at 60 s for scraping off the mixer bowl walls. Thereafter, the weighted amount of sand was added, and mixing was continued at 140 rpm for 120 s, and then, after scraping off the mixer bowl walls again, for another 120 s at 285 rpm.

After mixing, the produced mortars were cast and allowed to cure inside 160 mm × 40 mm × 40 mm molds at room conditions. During the mixing and casting processes, it was observed that the mortars sustained a level of workability and fluidity suitable for their use in construction applications. The specimens were demolded after at least one day or as soon as sufficient mechanical strength was achieved to withstand the demolding process. Thereafter, the specimens were allowed to continue curing at room conditions until the end of the seventh day after casting.

After 7 days of curing, the GPC mortar specimens were tested for unconfined compressive strength (CS) in a Instron 4505 universal mechanical testing machine (Instron, Norwood, MA, USA). A total of six specimens from each formulation were measured.

### 2.4. GPC Mortar Overall Composition

The composition of the six GPC mortar formulations, in terms of mass fractions (wt%) of each used material, is reported in Table 5. The first three formulations are single-precursor mortars (MK, BFA, and EAFS). The other three are multi-precursor formulations, with equal mass fractions of each used material in the precursor mix: 50% in the MK + BFA and MK + EAFS formulations; 33.3% in the MK + BFA + EAFS formulation.

## 3. Results and Discussion

### 3.1. Precursors Reactivity Tests

**Figure 5:**
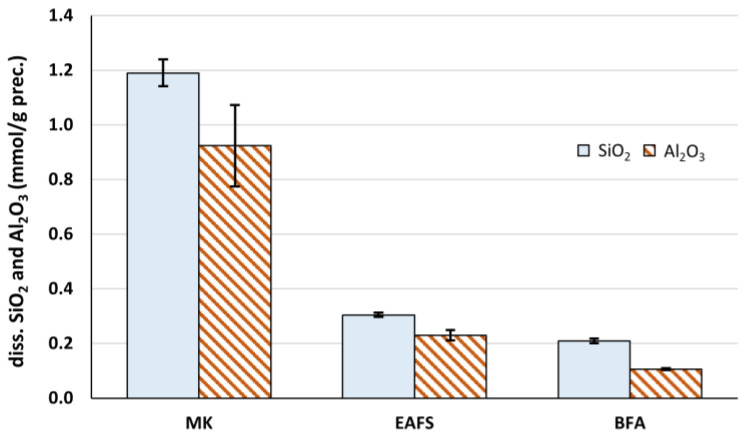
Dissolved amount of SiO_2_ and Al_2_O_3_ from each precursor, determined in the alkaline dissolution tests.

The potential reactivity of precursors towards the geopolymerization process was assessed by measuring the amount of dissolved aluminate and silicate species in a 10 M sodium hydroxide solution, as explained in Section 2.1. The results, in terms of mmol of SiO_2_ and Al_2_O_3_ per unit weight of precursor, are presented in Figure 5.

From Figure 5, it becomes clear that MK shows the highest aluminosilicate dissolution amount in the alkaline conditions used in this study: about 1.2 mmol of silicate and 0.9 mmol of aluminate per gram of material. Both EAFS and BFA show much lower amounts of dissolved aluminosilicates, below 0.3 mmol/g for SiO_2_ and Al_2_O_3_. This means that, in absolute terms, MK should be a better choice as a precursor for geopolymerization, when compared to BFA and EAFS, since it contributes with a higher amount of aluminosilicates to the formation of the GPC matrix.

**Figure 6:**
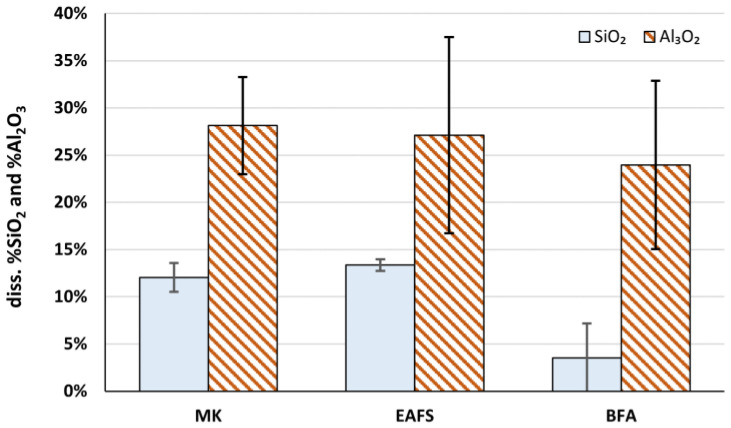
Dissolved SiO_2_ and Al_2_O_3_ reported as a fraction of the total amount measured by XRF.

On the other hand, we can also analyze the fraction of dissolved silicate and aluminate from each material (shown in Figure 6), assuming that the total content is that measured by XRF (see Table 2).

From this point of view, it can be concluded that the aluminate dissolution extent is in the same range for all three precursors, between 20 and 30% of the total content of this chemical species in the precursors, and the silicate dissolution extent is very similar in the case of MK and EAFS, about 12% to 13.5%. This means that the reactivity (in terms of alkaline dissolution) of the aluminate phase present in the solid material is very similar for all three precursors, and the same applies to the silicate phase in the case of MK and EAFS. Therefore, it seems that the significantly higher amount of dissolved silicates and aluminates from MK (Figure 5) mainly arises from the fact that the total content of aluminosilicate in its composition is also higher, since the reactivity of these phases in the solid raw material is similar among the different precursors.

### 3.2. Estimation of Dissolved Silicates and Aluminates in GPC Fresh Mortars 

**Figure 7:**
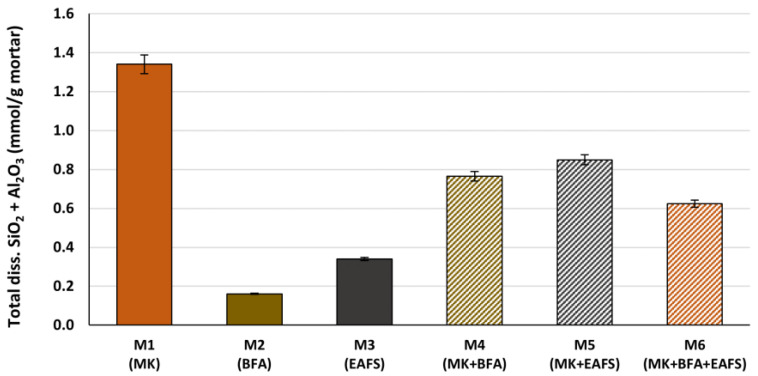
Estimated total dissolved SiO_2_ and Al_2_O_3_ for each GPC mortar formulation.

Based on the results of the dissolution tests with each precursor, the total amount of dissolved silicates and aluminates for each of the six GPC mortar formulations was estimated in terms of mmol SiO_2_ and Al_2_O_3_ per unit weight of fresh mortar and is shown in Figure 7.

For this estimation, the results of the dissolution tests were combined with the content of the respective precursor in the mortar formulation. In addition, the mass of the sodium silicate solution was also considered as it directly contributes to the total amount of dissolved silicates. The estimates in Figure 7 are therefore calculated for each mortar as follows:

$$\begin{array}{ll} Total\;diss.\;SiO_{2}\;+ & Al_{2}O_{3}\;\left(mmol/g\;mortar\right)\\ & =\underset{i}{\sum }\left[SiO_{2,i}\;(mmol/g\;P_{i})\times \%P_{i}\right]+\frac{0.267}{0.06008}\times \%SS\\ & +\underset{i}{\sum }\left[Al_{2}O_{3,\;i}\;(mmol/g\;P_{i})\times \%P_{i}\right] \end{array}$$

where SiO_2,*i*_ and Al_2_O_3,*i*_ are the amounts of dissolved silicates and aluminates as determined by the alkaline dissolution test for precursor *i* (MK, BFA or EAFS), %*P_i_* and %*SS* are the mass fractions of precursor *i* and sodium silicate solution in the mortar, respectively (reported in Table 5). The numerical parameter represents the concentration of SiO_2_ in the *SS* solution (in mmol SiO_2_/g *SS* solution).

From the data shown in Figure 7, it can be concluded that the formulation including MK as the only precursor (M1) produces the highest estimated amount of dissolved aluminosilicates in the fresh mortar. For the case of multi-precursor GPC mortar formulations, the M5 (MK + EAFS) formulation is estimated to produce a higher amount of dissolved aluminosilicates than the M4 (MK + BFA) formulation, since the content of MK is equal in both cases (13.6% of the total mortar weight) and, according to the dissolution tests, EAFS yields a slightly higher amount of dissolved aluminosilicates than BFA. In the case of the M6 (MK + BFA + EAFS) formulation, in which the content of MK is lower (9.1% of total mortar weight) when compared to M4 and M5 (13.6% of mortar weight), the estimated amount of dissolved aluminosilicates is also lower.

### 3.3. Mechanical Tests

**Figure 8:**
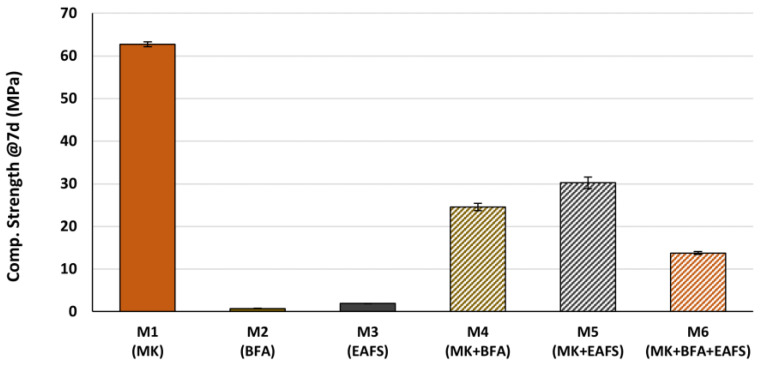
CS of GPC mortars after 7 days of curing.

After 7 days of curing, six specimens from each GPC mortar formulation were tested for unconfined CS. From the obtained results (shown in Figure 8), it can be stated that the trend of CS is very similar to that of estimated total dissolved SiO_2_ and Al_2_O_3_ presented in Figure 7 and leads, thus, to the conclusion that the amount of dissolved aluminosilicates plays a key role in terms of the mechanical performance of the GPC mortar.

**Figure 9:**
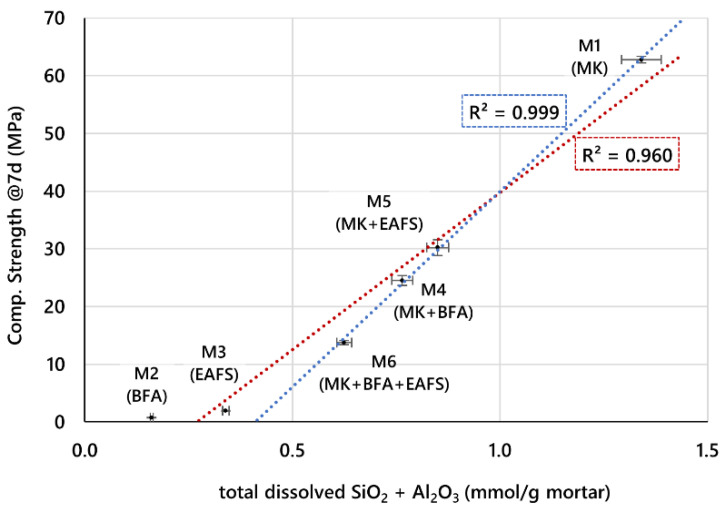
Correlation between CS of GPC mortars and total estimated dissolved aluminosilicates based on alkaline dissolution tests of precursors.

Furthermore, if the obtained CS is explicitly correlated with the estimated total amount of dissolved aluminosilicates in each formulation, the relationship becomes evident, as can be seen in Figure 9.

Considering all six formulations, it is clear that a higher estimated amount of dissolved SiO_2_ and Al_2_O_3_ corresponds to a higher CS of the mortar. The red dotted line is the best linear fit to the complete data set, obtained by the least squares fitting method, and, although it represents the general trend, some significant deviations are evident. On the other hand, if we only consider the GPC mortar formulations that include MK (excluding the M2 and M3 formulations), the linear fit (obtained by the same method) is very good (blue dotted line) and represents the data almost perfectly. From these results, it seems that, for estimated low amounts of dissolved SiO_2_ and Al_2_O_3_ (below about 0.6 mmol/g mortar) or CS below about 14 MPa, the influence of dissolved aluminosilicates in the development of strength is less significant. A possible explanation for this is that, for low CS (such as those obtained for the formulations M2 and M3, with BFA and EAFS as single precursors), other variables, such as the level of compacity of the materials and the mechanical strength of individual constituents of the mortar (disregarding the binding mechanism) become more important for the overall CS.

**Figure 10:**
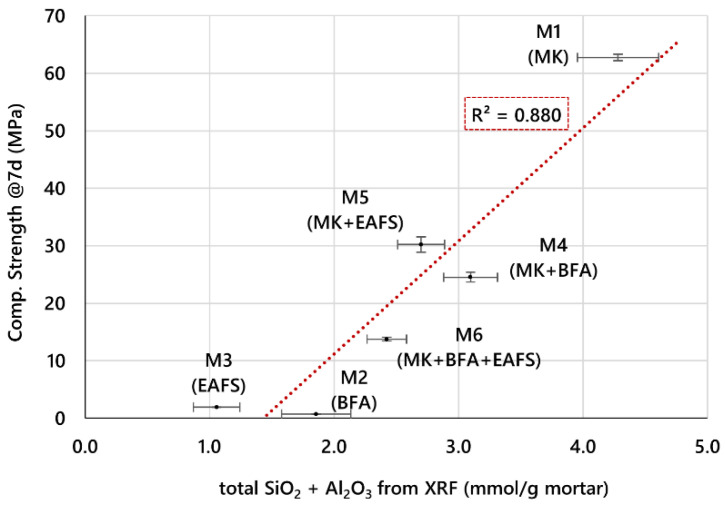
Correlation between CS of GPC mortars and total aluminosilicates based on precursor SiO_2_ and Al_3_O_2_ content as measured by XRF.

It is also worth comparing the correlation in Figure 9 to the one obtained if total precursor SiO_2_ and Al_3_O_2_ content, as measured by XRF, was used instead of the dissolved amounts from the alkaline dissolution test. For this case, the correlation with CS is shown in Figure 10.

As can be stated, although the data variation is still generally captured by a linear trend, there are significant deviations of experimental points from the best linear fit obtained by the least squares method. This fact makes this correlation much less valuable in predicting the CS of new formulations than the one using the estimated amounts of dissolved aluminosilicates in the fresh mortars based on the dissolution tests (shown in Figure 9). This is in line with the assumption that the actual amounts of aluminosilicates involved in the formation of the GPC matrix (which ultimately determines its mechanical properties) are more likely linked to the dissolved amounts of silicate and aluminate species in mortars (as estimated through dissolution tests) than to the total SiO_2_ and Al_2_O_3_ present in the precursors (as measured by XRF). Therefore, the potential of different raw materials for the formulation of GPC mortars seems to be better evaluated if the dissolution tests (proposed as a method for estimating the available aluminosilicates) are used, instead of simply relying on the precursors’ chemical composition normally obtained by XRF.

## 4. Conclusions and Future Work

The importance of studying the dissolution process of aluminosilicates from GPC precursors lies in the fact that it represents the basis for the formation of the GPC matrix, which will act as the binder that will enable the setting and hardening of the final material. In the present work, the alkaline dissolution process of MK, EAFS, and BFA was studied and correlated with the precursors’ composition and with the mechanical strength achieved by GPC mortar specimens prepared from these materials. The main conclusions are as follows:

- In the alkaline dissolution tests, MK produced a higher amount of dissolved SiO_2_ + Al_2_O_3_ (2.1 mmol/g) when compared to BFA and EAFS, which yielded 0.32 and 0.53 mmol/g, respectively. This was attributed to a higher content of silica and alumina in the composition of MK when compared to BFA and EAFS rather than to higher reactivity of these phases.
- The 7-day CS of the six GPC mortars prepared was shown to follow a linear correlation with the total dissolved aluminosilicates in the mortars, estimated by the results of the dissolution tests of precursors—the higher the amount of total dissolved aluminosilicates in the fresh mortar, the higher the CS achieved. The correlation based on the complete set of six different GPC mortars was found to yield an R^2^ of 0.96.
- Furthermore, if we consider only the four GPC mortar formulations with a total estimated amount of dissolved aluminosilicates above 0.6 mmol/g mortar, a very accurate linear correlation (R^2^ > 0.99) is obtained that explains the CS data in the range of around 15 MPa to 60 MPa.
- The same correlation based on the total silica and alumina content in precursors measured by XRF was shown to be much less accurate in explaining the CS variation (R^2^ ≈ 0.88). This is in line with the assumption that the amount of dissolved silicate and aluminate species has a stronger influence on the formation of the GPC matrix than the total content of SiO_2_ and Al_2_O_3_ in the source materials.
- These results support the usefulness of a simple method for screening and evaluating the relative potential of aluminosilicate-containing materials, in the present case inorganic industrial waste, in the geopolymerization process.

Further work will focus on assessing the applicability of the obtained correlation in the case of GPC mortars produced with different precursors and under similar conditions, so that a straightforward estimation of the CS of a generic GPC mortar could be achieved based on the reactivity of its constituent in a simple alkaline dissolution test. Additionally, along with the influence of SiO_2_ and Al_2_O_3_, the effect of the content of CaO in the precursors will also be studied since it is known to also contribute to the hardened properties of geopolymer materials.

## Figures and Tables

**Table 1:** PSD D10, D50, and D90 parameters for GPC precursors.

	D10 (μm)	D50 (μm)	D90 (μm)
MK	2.98	30.0	122
BFA	6.07	59.3	460
EAFS	0.80	8.43	113

**Table 2:** Main oxide composition of GPC precursors.

	SiO_2_	Al_2_O_3_	CaO	K_2_O	MgO	Fe_2_O_3_	MnO	SO_3_
MK	59.3%	33.5%	0.1%	3.2%	0.2%	3.2%	−	−
BFA	35.7%	4.5%	28.1%	4.1%	2.2%	1.3%	0.4%	3.2%
EAFS	13.7%	8.6%	24.0%	−	4.8%	24.6%	4.8%	0.5%

**Table 3:** PSD D10, D50, and D90 parameters for sand used as fine aggregates.

	D10 (μm)	D50 (μm)	D90 (μm)
sand	228	370	595

**Table 4:** Fixed geopolymerization ratios.

Ratio	Fixed Value
SiO_2_/Al_2_O_3_	4.4
H_2_O/Na_2_O	16
Water to solids (W/S)	0.15
Aggregates to precursors (Agg/P)	2.0

**Table 5:** Used GPC mortar formulations, expressed as mass fractions of each material.

Material	M1 (MK)	M2 (BFA)	M3 (EAFS)	M4 (MK + BFA)	M5 (MK + EAFS)	M6 (MK + BFA + EAFS)
**Precursor Mix**						
MK	26.6%	−	−	13.6%	13.6%	9.1%
BFA	−	27.9%	−	13.6%	−	9.1%
EAFS	−	−	27.7%	−	13.6%	9.1%
**Alkalination Mix**						
SS (aq.)	17.5%	1.6%	4.3%	9.8%	11.0%	8.0%
SH (aq., 32%)	1.2%	11.1%	10.2%	8.3%	7.4%	8.9%
SH (sol.)	1.4%	−	−	−	0.1%	0.9%
Water	−	3.5%	2.5%	0.2%	−	−
**Aggregates and Fillers**						
Sand	53.3%	55.8%	55.3%	54.5%	54.3%	54.8%

## Data Availability

Data is contained within the article.
